# A Lesser Known Side Effect of Tigecycline: Hypofibrinogenemia

**DOI:** 10.4274/tjh.2017.0310

**Published:** 2018-03-06

**Authors:** Fulya Yılmaz Duran, Halil Yıldırım, Emre Mehmet Şen

**Affiliations:** 1 Bozyaka Training and Research Hospital, Clinic of Anesthesiology and Reanimation, İzmir, Turkey

**Keywords:** Hypofibrinogenemia, Tigecycline, Hemoglobine level

## To the Editor,

Fibrinogen is a soluble protein that is produced in hepatocytes. It participates in blood coagulation and is considered as an acute phase protein with a half-life of 3 to 4 days [1,2]. Fibrinogen values range from 200 to 400 mg/dL [1,3]. While hyperfibrinogenemia is correlated with systemic inflammation and malignancy, hypofibrinogenemia can be observed in chronic inherited diseases, acquired hepatic dysfunction, severe malnutrition, disseminated intravascular coagulation, abnormal fibrinolysis, large volumes of blood transfusions, and drug administration [1,3,4].

Tigecycline is the first member of the glycylcyclines. This is a new class of drugs structurally similar to tetracyclines [1,5,6]. It can be used to treat complicated intraabdominal infections, complicated skin infections, and community-acquired bacterial pneumonia [6,7]. Tigecycline inhibits protein synthesis by binding to the 30S ribosomal subunit and blocking entry of aminoacyl-transfer RNA molecules into the A side of the ribosome [1,2]. It has poor bioavailability and so requires intravenous administration with a loading dose of 100 mg, followed by 50 mg twice daily [1,6]. In patients with child C cirrhosis, the manufacturer suggests a reduced dose (25 mg twice daily) [5]. Multiple adverse events have been reported [1]. A decrease in fibrinogen levels has been observed and severe coagulopathy has also been reported during tigecycline treatment [2,6]. 

A 90-year-old female patient was admitted to the emergency department with the complaint of nausea and vomiting for 3 days. Her medical history included asthma and chronic renal failure. Physical examination revealed respiratory failure, unconsciousness, and bilateral rhonchi on chest auscultation. Computer tomography of the thorax revealed bilateral effusion, consolidation, and diaphragm hernia. She was intubated and transferred to the intensive care unit (ICU). The initial antiinfective regime consisted of piperacillin/tazobactam at 3x4.5 g and ciprofloxacin at 2x400 mg intravenously. On the 15^th^ day of admission, the antibiotherapy was switched to tigecycline because of unresponsiveness to the first antibiotherapy.

On the 10^th^ day of tigecycline therapy, a progressive worsening of hyperbilirubinemia was noted. Simultaneously, the hemoglobin level was markedly decreased (Table 1). To exclude hepatic or biliary pathology and abdominal pathology, abdominal ultrasonography was performed, followed by computed tomography, but they revealed no pathological entities. Moreover, fibrinogen was lower. As we suspected an association with the use of tigecycline, we discontinued the drug on the 10^th^ day of therapy. After discontinuation of tigecycline, fibrinogen levels improved markedly within 8 days and bilirubin levels tended to be lower. On the 40^th^ day of ICU admission, she died.

We hypothesized that the decrease in fibrinogen level was a side effect of tigecycline because hypofibrinogenemia became apparent only after 10 days of antimicrobial therapy and the fibrinogen level increased after the withdrawal of tigecycline. Life-threatening coagulopathy and hypofibrinogenemia cases induced by tigecycline were reported by Rossitto et al. [5], Pieringer et al. [7], and Sabanis et al. [1]; clinical studies were reported by Routsi et al. [6] and Zhang et al. [2] in the literature. However, the main mechanism by which tigecycline provokes hypofibrinogenemia is ambiguous [1,5]. It could be by intestinal microflora or by hepatic dysfunction [1,5,7]. The posttranscriptional regulation of the fibrinogen gene by miRNAs could be the cornerstone in this field [1]. 

We suggest routine strict monitoring of coagulation parameters in patients receiving tigecycline. If patients develop hypofibrinogenemia, one should consider discontinuation of the drug.

## Figures and Tables

**Table 1:**
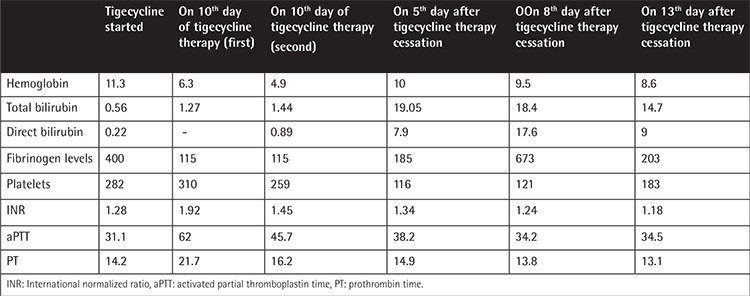
Laboratory findings of the patient.
